# Exploring Tactile Perceptual Dimensions Using Materials Associated with Sensory Vocabulary

**DOI:** 10.3389/fpsyg.2017.00569

**Published:** 2017-04-13

**Authors:** Maki Sakamoto, Junji Watanabe

**Affiliations:** ^1^Department of Informatics, The University of Electro-CommunicationsTokyo, Japan; ^2^NTT Communication Science Laboratories, Nippon Telegraph and Telephone CorporationKanagawa, Japan

**Keywords:** tactile materials, tactile perceptual dimensions, sound symbolic words, affective evaluation, friction

## Abstract

Considering tactile sensation when designing products is important because the decision to purchase often depends on how products feel. Numerous psychophysical studies have attempted to identify important factors that describe tactile perceptions. However, the numbers and types of major tactile dimensions reported in previous studies have varied because of differences in materials used across experiments. To obtain a more complete picture of perceptual space with regard to touch, our study focuses on using vocabulary that expresses tactile sensations as a guiding principle for collecting material samples because these types of words are expected to cover all the basic categories within tactile perceptual space. We collected 120 materials based on a variety of Japanese sound-symbolic words for tactile sensations, and used the materials to examine tactile perceptual dimensions and their associations with affective evaluations. Analysis revealed six major dimensions: “Affective evaluation and Friction,” “Compliance,” “Surface,” “Volume,” “Temperature,” and “Naturalness.” These dimensions include four factors that previous studies have regarded as fundamental, as well as two new factors: “Volume” and “Naturalness.” Additionally, we showed that “Affective evaluation” is more closely related to the “Friction” component (slipperiness and dryness) than to other tactile perceptual features. Our study demonstrates that using vocabulary could be an effective method for selecting material samples to explore tactile perceptual space.

## Introduction

The sense of touch is a growing interest in the fields of product design (Citrin et al., 2003; Peck and Childers, 2003; Grohmann et al., 2007), clothing (Na and Kim, 2001; Workman, 2010; Rahman, 2012), and cosmetics (Barnes et al., 2004; Nakatani et al., 2013). Specifically, it is relevant for designing products because the feel of products when handled can greatly affect a customer's final decision to purchase the product (Choi and Jun, 2007). Indeed, affective attachment experienced during tactile exploration has been recognized as a strong driver of product preference (Millar and Millar, 1996; Schifferstein, 2006; Sonneveld and Schifferstein, 2008; Peck and Shu, 2009). For designers, grasping the relationship between surface attributes and the psychological responses from customers is critical for designing the surface texture of a product.

Psychophysical studies contain a large body of literature regarding the dimensions of tactile perception (Yoshida, 1968; Lyne et al., 1984; Hollins et al., 1993, 2000; Picard et al., 2003; Gescheider et al., 2005; Bergmann Tiest and Kappers, 2006; Yoshioka et al., 2007; Chen et al., 2009; Guest et al., 2012; Okamoto et al., 2013). However, how tactile perceptual space is determined and how it is related to affective evaluations are still central questions both in perception psychology and in design applications that rely on touch. A critical problem affecting the results of past studies has been the criteria for selecting material samples. Most studies have tested only a limited number of material samples in specific areas (e.g., manufacturing products or textured fabrics). In fact, the numbers and types of major tactile dimensions reported in past studies have differed depending on the material samples used in the experiments (see Okamoto et al., 2013 for a review). In product design, although material has been a central point of research and practice for decades (Manzini, 1986; Ashby and Johnson, 2009; see Karana et al., 2015 for more details), as Karana et al. (2015) points out, a systematic method for defining and designing material experiences is lacking. This is likely because most studies take a particular material as a starting point and explore its engineering properties for potential use in a product.

The current study revisits tactile perceptual dimensions and their associations with affective evaluations by exploring tactile perceptual space using a variety of material samples that were selected with a different guiding principle. In contrast to the Material Driven Design Method proposed by Karana et al. (2015), which starts with a tangible material to facilitate the design process, our method begins with structuring tactile perceptual space. To explore as broad a tactile perceptual space as possible, we focused on sensory vocabulary called “sound symbolic words” (SSWs) as a guiding principle, and collected a varied sample of materials according to SSWs related to touch. Having detailed and reliable vocabulary is important for meaningful descriptions of perceptual experiences (Osgood, 1952; Bhushan et al., 1997; Guest et al., 2011), and we hypothesized that analyzing sensory vocabulary could be an effective way to investigate perceptual space (Malt and Majid, 2013). SSWs are adjective-like words that have associations between sound and meaning. The existence of SSWs has been demonstrated in a wide variety of languages (Köhler, 1929; Sapir, 1929; Bolinger, 1950; Hinton et al., 1994; Nuckolls, 1999; Ramachandran and Hubbard, 2001; Schmidtke et al., 2014). For example, English words starting with “sl-” such as “slime,” “slush,” “slop,” “slobber,” “slip,” and “slide” symbolize something smooth or wet (Bloomfield, 1933). Semantic distinctions between tactile SSWs called “ideophones” have been reported to express tactile sensations in detail in Gbeya (a language of the Central African Republic; Samarin, 1967), and reproduce salient psychophysical dimensions in Siwu (a language of Ghana; Dingemanse and Majid, 2012). Japanese has a lexical class of SSWs called “mimetics” (Kita, 1997) or “onomatopoeia” that are used to express vivid sensations in everyday life (e.g., “sara-sara” represents a dry and smooth sensation like hair, and “zara-zara” represents a dry and rough sensation like sand paper). Recently, there has been a growing interest in using SSWs to explore perceptual space. Doizaki et al. (2016) proposed a system that automatically estimates multidimensional ratings of touch from a single SSW that is spontaneously and intuitively expressed by a user, and visualizes tactile perceptual space using the estimated SSW ratings. Their study provides an alternative method for estimating the fine quality of tactile sensations. SSWs can be used to examine other perceptual spaces as well. Sakamoto and Watanabe (2015) investigated sound symbolism in taste by analyzing SSWs spontaneously and intuitively used by participants to express tastes/textures and showed that SSWs could be important indexes for investigating differing levels of gustatory perceptual dimensions, including emotional evaluations like pleasant/unpleasant.

In this paper, we therefore focused on Japanese SSWs to investigate tactile perceptual dimensions. Japanese has a large number of SSWs for expressing touch sensations, and the vocabulary constitutes a linguistic system with rigid phonological constraints and a strong systematic association between form and meaning that is constituted socially (Hamano, 1998). For example, Japanese words that express a sense of smoothness often use the consonant /s/ in the first syllable as in “sara-sara” and “sube-sube,” while those expressing roughness often use /j/ or /z/ in the first syllable, as in “jari-jari,” “jori-jori,” and “zara-zara” (Watanabe et al., 2012). Japanese SSWs generally have a fixed structure in which a combination of two syllables is repeated in the form of CVCV-CVCV (C, consonant; V, Vowel). New SSWs can be created only by combining phonemes. The variation of syllables (especially the first syllable) is expected to correspond to distinct sensory categories (Hamano, 1998), and we should be able to systematically explore tactile perceptual space by encompassing materials associated with all Japanese phonemes that are located at the beginning of the words, at least to the extent that the perceptual space is one that the Japanese language can express. We therefore regarded Japanese SSWs as a useful index that can cover a wide range within tactile perceptual space, and used them to collect a variety of tactile material samples.

Our study differs critically from previous studies on tactile perceptual dimensions in that the criteria for collecting material samples is based on sensory vocabulary, not on physical properties, with tactile perceptual space approached from a phenomenological perspective (perceptual classification and its labeling), rather than a physical one. Phenomenology is mostly used to provide a better understanding of the way people perceive the world, and phenomenological methods have been introduced in fields of design that aim at providing design-relevant knowledge on how to design new artifacts (see Deckers et al., 2012). In this study, we chose 120 material samples corresponding to Japanese SSWs for expressing tactile sensations with a systematic association between sounds and meanings. Using the 120 materials, we performed psychophysical experiments to understand tactile perceptual dimensions and their associations with affective evaluations.

## Materials and methods

### Ethics statement

This study was approved by the Ethics Committee of the University of Electro-Communications, Tokyo, Japan, and adhered to the tenets of the 1964 Declaration of Helsinki (most recently amended in 2008). All participants provided written informed consent before the experiments. Experimental protocols and written informed consent were presented to the ethics committee.

### SSWs as an index for collecting tactile materials

We generated Japanese SSWs to be used as an index for collecting tactile material samples that represented as large a tactile perceptual space as possible. Initially, we made SSW-like expressions by combining all Japanese syllables. We created SSW-like expressions in the typical two-syllable-repeated form (e.g., “saka-saka,” “saki-saki,” “saku-saku,” “sake-sake,” and “sako-sako”) and added all types of special phonemes used in Japanese SSWs (syllabic nasals /N/, choked sounds /Q/, long vowels /R/, and adverbs ending in /Li/). From this considerably large number of SSW-like expressions (14,584), three 21-year old native Japanese speakers (two male and one female) who use SSWs in daily life selected words that would be acceptable as Japanese tactile SSWs. One-hundred and ten of these SSW expressions were chosen by two or three speakers and their degree of conventionality was tested using a Google search on June 6, 2014. As a result, more than 100,000 search results were obtained for all of the 110 SSWs. The first syllables of the 110 words covered all Japanese consonants except /l/, which is never used as the first syllable in conventional tactile SSWs, indicating that we can systematically explore tactile perceptual space by collecting materials associated with the first syllables.

To analyze the relationships among the 110 SSWs, we used a system that can convert a tactile SSW into rating scores for multiple touch-related adjectives. In a previous study (Doizaki et al., 2016), we built a database of sound symbolic associations between SSW phonemes and their sensory impressions. The database utilizes 26 pairs of opposing adjectives (e.g., hard/soft, wet/dry) that were selected based on the results of separate psychological experiments in which participants evaluated impressions of SSWs. Using this database, here we estimated a given SSW's ratings for each of the 26 pairs adjectives by averaging the impressions given in the database for each phoneme of the SSW. To intuitively grasp the relationships between the 110 SSWs, we performed a principal component analysis of the 110 SSWs using scores for 6 of the 26 pairs (“hard/soft,” “rough/smooth,” “bumpy/flat,” “sticky/slippery,” “wet/dry,” and “warm/cold”), which constitute the basic tactile dimensions (see Okamoto et al., 2013). Then, we generated a distribution diagram of the SSWs using the first and second principal components as the horizontal and vertical axes, respectively.

### Collecting materials

Five experts in linguistics and psychology who have experience in tactile psychophysical experiments (including the two authors) selected 120 tactile materials whose textures and properties could be expressed by one or more of the 110 SSWs. Thus, the 110 SSWs were assigned to the 120 materials. These assignments were then confirmed by four additional naïve participants.

### Evaluating subjective impression

Sixty naïve participants (30 males and 30 females; age range, 19–26 years) took part in the experiments. All were native Japanese speakers, unaware of the purpose of the experiments, and none had any known abnormalities in verbal or tactile sensory systems or in any particular skills with respect to touch. They visited a laboratory at the University of Electro-Communications for 1 day to take part in the experiment.

**Figure 1:**
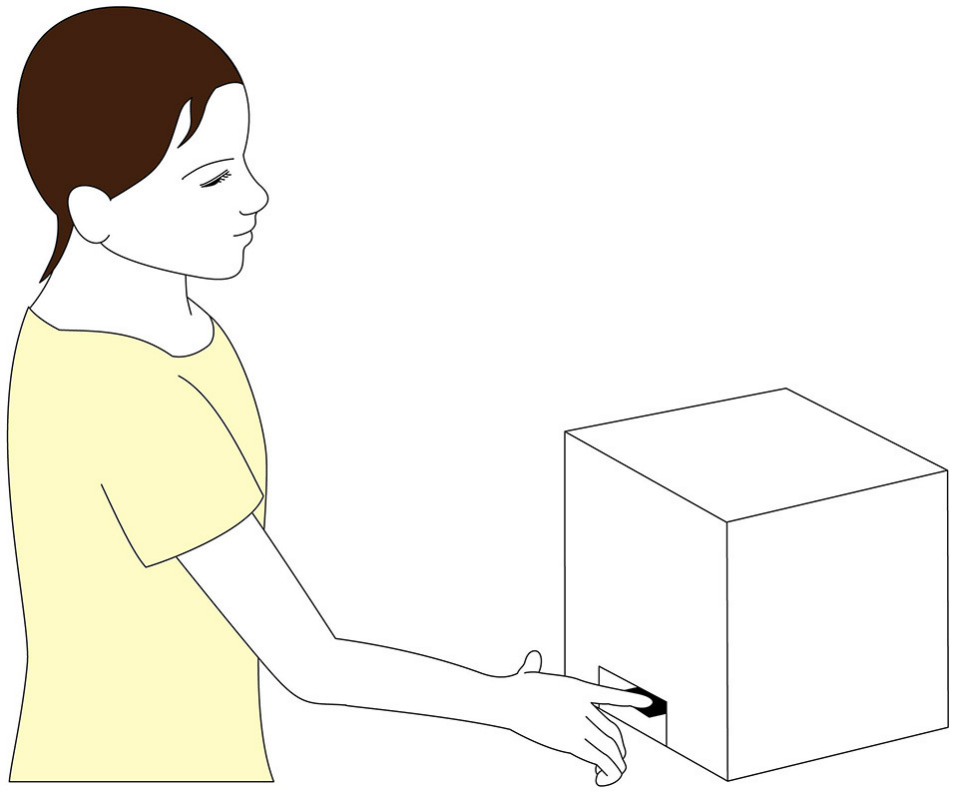
**Illustration of the experimental setup**.

The 60 participants were randomly classified into two groups (*n* = 30 for each) to reduce the number of materials each participant had to touch. Sixty materials were assigned to each group. Participants sat in front of a box with an 8 × 10 cm opening (the material box), and touched a material through the opening using the index finger of their dominant hand. Participants could not see the material while they were touching, and were asked at this time to rate the material on each of the 26 pairs of adjectives (Table 1). Figure 1 shows an illustration of the experimental setup.

**Table 1:** **List of 26 adjective pairs**.

**Basic tactile evaluation**	**Material-oriented evaluation**	**Affective evaluation**
Smooth—Rough	Elastic—Non-elastic	Comfortable—Uncomfortable
Bumpy—Flat	Firm—Fragile	Relieved—Uneasy
Hard—Soft	Regular—Irregular	Good—Bad
Warm—Cold	Repulsive—Non-repulsive	Impressive—Unimpressive
Slippery—Sticky	Sharp—Dull	Luxury—Cheap
Wet—Dry	Clean—Dirty	Pleasant—Irritating
	Stretchy—Non-stretchy	Familiar—Unfamiliar
	Thick—Thin	Eccentric—Ordinary
	Heavy—Light	Natural—Artificial
	Strong—Weak	Intense—Calm

The six pairs of adjectives in the left column are those given as basic tactile dimensions (Okamoto et al., 2013). The other 20 pairs of adjectives are related to material-oriented and affective evaluations. Most of the 26 pairs of adjectives are included in the 262-word English vocabulary for expressing tactile sensations (Guest et al., 2011). Ratings were made on a seven-point Likert-like scale (e.g., −3 = very smooth, 3 = very rough). Participants could freely run their fingers along the surface or press their finger into the material to ascertain a variety of material properties (Lederman and Klatzky, 1987). No time limit was given for rating. The experimenter placed one material in the material box, and only replaced it with the next material after the participant had touched and rated it. The materials were presented in random order. The experiment took about 50–60 min for each participant.

## Results

### SSWs as an index for collecting tactile materials

**Figure 2:**
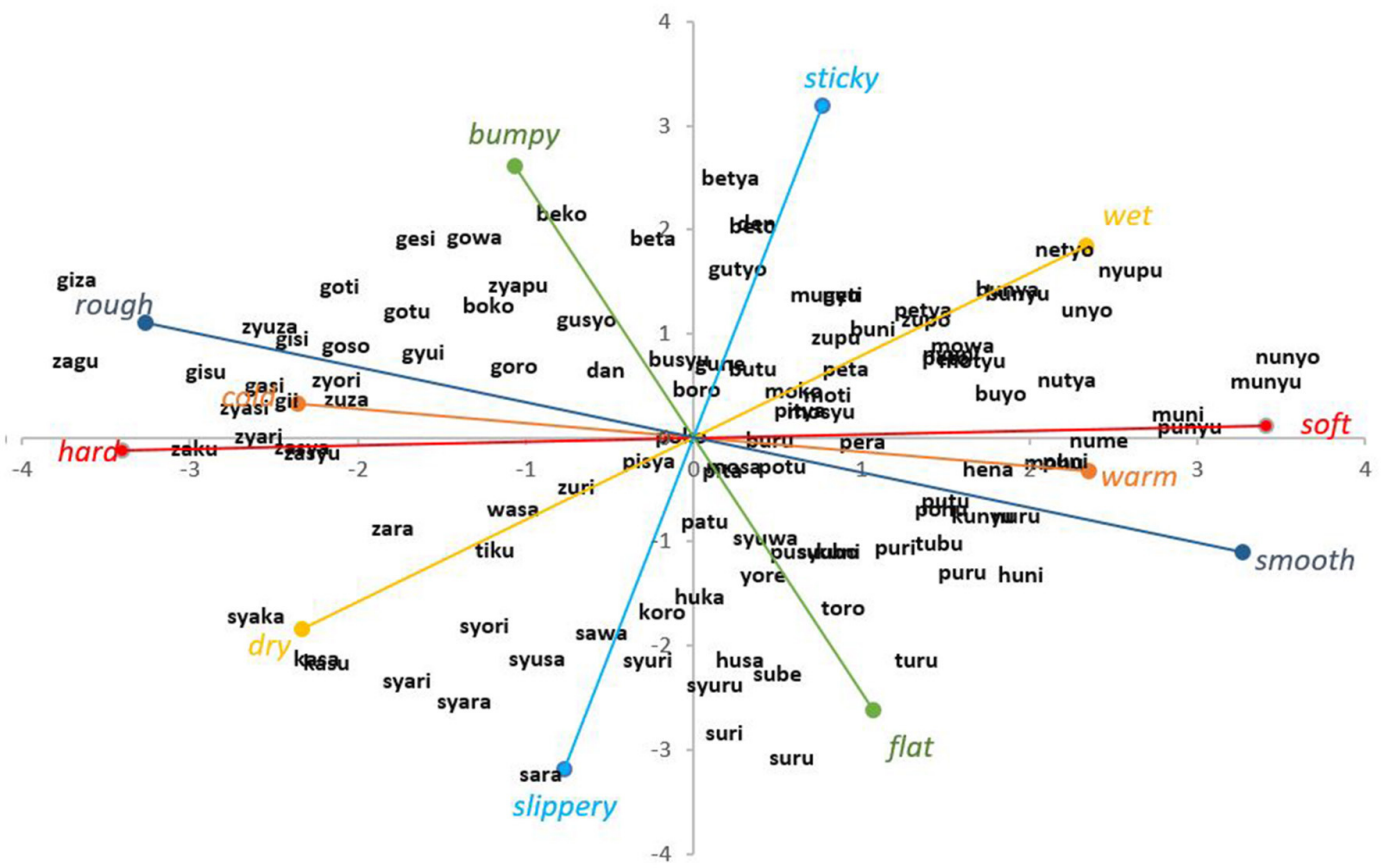
**Scatter plot of 110 identified SSWs**. The repetition of syllables in the SSWs are omitted (CVCV-CVCV is expressed as CVCV). *SSW*, sound symbolic word; *C*, consonant; *V*, Vowel.

Figure 2 is the scatter plot of the 110 SSWs based on the first and second principal components as the horizontal and vertical axes, respectively. The cumulative contribution ratio of the first and second principal components was 80.0%. We have added the six pairs of basic tactile adjectives to the diagram based on the principal component loadings. Additionally, SSWs expressing closely related sensations are located close to each other on the distribution diagram. The diagram is thus a spatial map that reveals of how tactile sensations are categorized by Japanese language.

### Material collection

**Figure 3:**
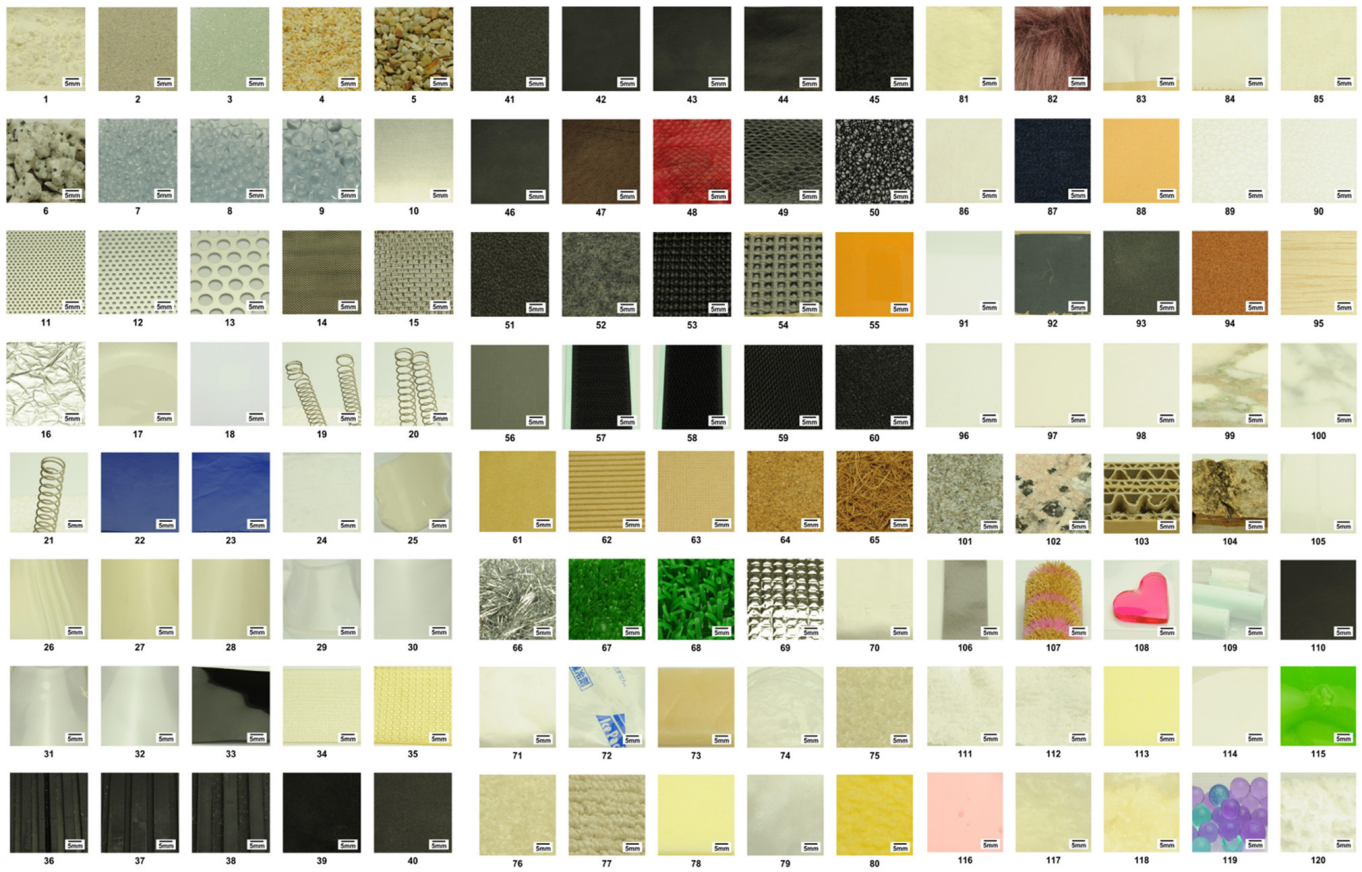
**Images of the 120 materials used in the experiment**.

The 120 materials are listed with their compositions and the associated SSWs in Appendix in Supplementary Material. Images of the materials, such as particle, metal, ceramic, glass, spring, elastomer, soft-urethane, gel, rubber, leather, polyurethane, paper, fabric, clay, polystyrene, rough paper, stone, and others are given in Figure 3. Most of the material samples were cut into 6 × 6 cm squares and stacked into 2-mm thick layers. The rocks and sand were placed loose in a container.

### Evaluating subjective impressions

Each of 30 participants gave 26 ratings (one for each pair of adjectives) for half of the 120 material samples and the other 30 participants did the same for the other half of the samples. We conducted a factor analysis to detect basic structures in the relationships among adjectives and to classify them. More specifically, principal component analysis was applied to mean values of the 26 adjective ratings. This type of factor analysis is a common statistical method for describing variables via the lowest number of unobserved variables called factors. Although this method may simply cluster similar categorical words together and depends on the words pairs selected for the analysis, it remains useful and has been employed in numerous psychophysical studies, including tactile studies (see a review by Okamoto et al., 2013). Results of the analysis are shown in Table 2.

**Table 2:** **Factor loadings for the 26 adjective pairs**.

**Adjective pairs**	**Factor**					
	**1**	**2**	**3**	**4**	**5**	**6**
Comfortable—Uncomfortable	0.95	−0.01	0.12	−0.03	0.12	−0.09
Good—Bad	0.95	−0.04	0.13	0.05	0.08	−0.04
Pleasant—Irritating	0.89	0.30	0.11	−0.08	−0.09	−0.11
Clean—Dirty	0.89	0.28	0.15	−0.07	−0.09	0.21
Relieved—Uneasy	0.89	−0.04	0.21	−0.04	0.32	−0.15
Familiar—Unfamiliar	0.89	−0.16	0.10	−0.09	0.22	−0.17
Slippery—Sticky	0.80	0.46	−0.17	−0.07	−0.16	−0.01
Repulsive—Non-repulsive	−0.71	−0.23	−0.18	0.23	0.40	0.25
Ordinary—Eccentric	0.64	0.43	0.35	−0.15	0.26	−0.14
Calm—Intense	0.61	−0.40	0.55	−0.08	0.08	−0.31
Impressive—Unimpressive	−0.57	−0.28	−0.44	0.35	−0.38	0.09
Wet—Dry	−0.57	−0.49	0.39	0.26	−0.34	−0.04
Hard—Soft	0.06	0.92	−0.10	0.20	−0.08	0.12
Elastic—Non-elastic	−0.30	−0.87	−0.07	0.13	0.15	0.24
Stretchy—Non-stretchy	−0.30	−0.86	0.06	0.08	0.01	0.08
Strong—Weak	−0.19	0.73	0.05	0.57	0.09	0.26
Sharp—Dull	−0.02	0.69	−0.39	−0.07	−0.16	0.27
Firm—Fragile	0.00	0.66	0.30	0.53	0.15	0.28
Bumpy—Flat	−0.13	0.08	−0.88	0.02	−0.01	−0.08
Smooth—Rough	0.26	−0.31	0.76	0.10	−0.36	0.06
Regular—Irregular	0.23	0.29	0.62	−0.06	0.09	0.56
Heavy—Light	−0.26	0.24	0.16	0.86	−0.25	−0.02
Thick—Thin	−0.05	−0.21	−0.28	0.74	−0.02	−0.05
Luxury—Cheap	0.46	0.16	0.35	0.49	−0.26	−0.09
Warm—Cold	0.35	−0.27	−0.13	−0.31	0.71	−0.03
Natural—Artificial	0.38	−0.03	−0.08	0.00	0.04	−0.70
Eigen value	9.95	5.48	3.42	2.14	1.46	1.01
Contribution ratio	38.26	59.35	72.49	80.71	86.34	90.22

Six factors were identified, which we call “Affective evaluation and Friction,” “Compliance,” “Surface,” “Volume,” “Temperature,” and “Naturalness.”

We selected factors having an eigenvalue >1.0 and confirmed that the first six factors accounted for 91.0% of the total variance. Factor 1 was an “Affective evaluation” dimension, with high loadings on scales for Comfortable/Uncomfortable, Good/Bad, Pleasant/Irritating, Clean/Dirty, Relieved/Uneasy, Familiar/Unfamiliar, Ordinary/Eccentric, Calm/Intense, and Impressive/Unimpressive. Factor 1 also included the “Friction” components of Slippery/Sticky, Repulsive/Non-repulsive, and Wet/Dry such that positive affective evaluations were strongly associated with non-frictional features such as slipperiness, non-resistance, and dryness. Factor 2 was a “Compliance” dimension, with high loadings on scales for Hard/Soft, Elastic/Non-elastic, Stretchy/Non-stretchy, Sharp/Dull, and Firm/Fragile. This dimension also included the adjective pair Strong/Weak. It is reasonable that a hard, non-elastic, non-stretchy, sharp, and firm object is perceived as strong. Factor 3 was a “Surface” dimension, with high loadings on Bumpy/Flat, Smooth/Rough, and Regular/Irregular. These adjective pairs are related to surface texture or geometry. Factor 4 was a “Volume” dimension, with high loadings on Heavy/Light, and Thick/Thin. This dimension is a shape property that was independent of the material properties. This property has not been addressed in previous studies, most of which focused on material properties and either only allowed participants to touch material surfaces or only considered flat materials (Okamoto et al., 2013). The adjective pair Luxury/Cheap also had a high loading for this dimension, although it was also related to the “Affective evaluation and Friction” dimension. Factor 5 was a “Temperature” dimension with a high loading for the scale Warm/Cold. Factor 6 was a “Naturalness” dimension, with a high loading for the Natural/Artificial scale. This dimension has not been highlighted in any previous study.

## Discussion

Our study collected 120 material samples based on 110 sensory words related to tactile sensations. Results yielded six major dimensions for tactile perceptions and evaluations, which included almost all perceptual factors mentioned in other studies (Okamoto et al., 2013), plus several additional ones. This suggests that our vocabulary-based material collection covered a wider range of perceptual space compared with commonly used physical-property-based collection methods, although the arbitrariness of connections between words and materials might not be negligible. The novel point of this study is that the tactile perceptual space was approached from a phenomenological viewpoint rather than from a physical viewpoint, and was tested in a psychophysical experiment. We believe that the combination of material collection using language and its psychophysical validation can contribute to revealing a representation of human engagement with the tactile world.

We focused on sensory vocabulary for collecting material samples, and specifically on SSWs as opposed to normal adjectives. One SSW expresses rich and delicate information that must be otherwise expressed by combinations of two or more normal adjectives. Additionally, there are a larger number of SSWs than normal adjectives in Japanese for expressing tactile sensations. This suggests that Japanese SSWs describe a wider range of tactile sensations than normal Japanese adjectives. In fact, Sakamoto and Watanabe (2013) showed that more than 20 Japanese SSWs were evoked by the tactile sensations for 40 materials, while only 15 normal Japanese adjectives were evoked for the same materials. Moreover, there is a systematic and strong association between the first syllable of Japanese SSWs and the category of tactile sensation (Watanabe et al., 2012). Further, SSWs might describe sensory qualities with a finer resolution given that different types of SSWs were associated with imitation metals and real metals (Sakamoto et al., 2016). Here, we therefore used Japanese phonemes to express tactile experience as an index for collecting material samples.

The main result of the current study is that the 26 pairs of adjectives could be described by six factors. This result is not simply a measure of the conceptual closeness of the adjectives that describe the tactile features, but is also a measure of the co-occurrence of tactile dimensions among the 120 materials in terms of the 26 adjective pairs. Because we collected materials based on sensory vocabulary, the nature of the materials varied considerably and the six factors reflect a broad range of perceptual dimensions. Although we cannot overcome the limitations of vocabulary or exclude bias of linguistic descriptions, our method thus provides benefits over those used in previous studies.

Fundamental tactile dimensions obtained in previous studies did not completely match what we found here, but were somehow complementary (see Okamoto et al., 2013). We found that the six dimensions “Affective evaluation and Friction,” “Compliance,” “Surface,” “Volume,” “Temperature,” and “Naturalness” are dominant factors in human tactile perceptual experience. The three perceptual dimensions (“Compliance,” “Surface,” and “Temperature”) have been frequently observed in other studies, with “Compliance” and “Surface” being regarded as robust (Hollins et al., 1993, 2000; Bergmann Tiest and Kappers, 2006), and thermal information being known to play a critical role in material perception (Ho and Jones, 2006; Tiest and Kappers, 2009; Tiest, 2010). Note that the “Surface” dimension could be further divided into sub-dimensions (coarse and fine) in terms of corresponding sensory channels (Hollins and Rinser, 2000), and that the “Temperature” dimension is unique in terms of its functional connectivity in the brain because temperature information is processed primarily through the insular cortex, not primary somatosensory cortex (Peltz et al., 2011). Given that the perceptual characteristic of these three dimensions are obtained through different movements (pushing, tracing, and static touch; Lederman and Klatzky, 1987), regarding them as essential and independent dimensions might be reasonable.

The first dimension extracted in our study, “Affective evaluation and Friction,” includes almost all affective evaluations and a few perceptual adjectives. Objects and their meanings that are evaluated using multiple adjectives are known to be categorized primarily with affective criterion (Sakamoto and Utsumi, 2014). This is consistent with our finding that affective evaluations are included in the primary dimension. The first dimension also included adjective pairs pertaining to frictional perceptions (Slippery/Sticky and Repulsive/Non-repulsive) and moisture (Wet/Dry). Interestingly, Slippery/Sticky and Wet/Dry have been obtained as important tactile factors in several other studies, although they have never been extracted together in a single experiment (Okamoto et al., 2013). Additionally, although evaluations of friction and roughness seem to share some underlying mechanisms (Smith et al., 2002), given that roughness was insensitive to the frictional status of the surfaces (Taylor and Lederman, 1975; Nonomura et al., 2009; Skedung et al., 2011), the frictional evaluations in the first dimension appear to be independent of the “Surface” dimension.

Little is known about the relationship between affective evaluations and perceptual factors (Essick et al., 2010), although studies on single perceptual properties—mostly on roughness (Choi and Jun, 2007; Kitada et al., 2012)—have been performed. Studies on multiple tactile properties have indicated that users prefer smooth sandpaper, cardboard, and paper (seven materials; Ekman et al., 1965), slippery and hard cardboard, flexible materials, and laminate boards (37 materials; Chen et al., 2009), smooth and soft materials encountered in everyday life (48 materials; Klöcker et al., 2012), and soft and smooth virtual surfaces, where only roughness and compliance were varied (Hilsenrat and Reiner, 2011). In our study, crystalline limestone (#100) and the ceramic dish (#17) were highly associated with evaluations of “Slippery” and “Comfortable,” while ether-based polyurethane (#23) and methylcellulose (#117) were highly associated with “Sticky” and “Uncomfortable.”

The association between “sticky” and “uncomfortable” has been reported in studies examining the feeling of disgust, with mushiness, stickiness, and sliminess being related to feelings of disgust from the viewpoint of pathogen avoidance (Curtis and Biran, 2001). Additionally, experiments have shown that wet stimuli and stimuli resembling biological consistencies (stickiness) are evaluated as more disgusting (Oum et al., 2011). Here, we also extracted factors pertaining to cognitive evaluations (Good/Bad) in the first dimension. Three functions—evolutional (pathogen avoidance), biological (mate choice), and social (moral judgment)—have been suggested to be deeply related to feelings of disgust (Tybur et al., 2009). Given that stickiness is related to a feeling of disgust within a biological context (Clean/Dirty), feeling Good/Bad could be included in the same dimension.

The fourth dimension “Volume” is related to aspects of an object's shape, such as thickness (Thick/Thin), weight (Heavy/Light), and its valuation (Luxury/Cheap). For example, crystalline limestone (#99) and gray granite (#101) were evaluated as thick, heavy, and expensive objects, while polypropylene film (#106) and denim (#87) were evaluated as thin, light, and cheap. Although valuation is deeply related to touch (for example, increased duration of physical contact leads to higher valuations in a manner similar to the duration of actual ownership (Wolf et al., 2008), little attention has been paid to valuation in touch research. Our interpretation of the fourth dimension is that thicker and heavier objects tend to be perceived as more luxurious, although valuation is also related to the “Affective evaluation and Friction” dimension. Considering the relationship between size and perceived quality (e.g., Yan et al., 2014), this interpretation is reasonable.

Analysis revealed “Naturalness” as a sixth major dimension of tactile evaluations. The concept of naturalness has been used widely in studies of sensory perception, but the meaning is somehow ambiguous and an objective definition has been lacking. In this study, we defined naturalness as “the feeling that an object is something derived from nature” (Overvliet and Soto-Faraco, 2011), and asked participants to rate “Naturalness” on a seven-point scale ranging from “very natural” to “very artificial.” Objects that were rated the most natural were granite (#102) and water (#114), which are natural materials, while objects rated the most artificial were aluminum product (#13) and a stainless-steel coil spring (#21). The feeling of naturalness is likely to be assessed from information gathered by multiple modalities, which can provide complementary knowledge about materials. For example, the contributions of vision and touch have been experimentally validated in perceiving the naturalness of wood (Overvliet and Soto-Faraco, 2011). Our results (Table 2) indicate that the “Regular/Irregular” categorization within the “Surface” dimension, and “Elastic/Non-elastic” in the “Compliance” dimension are also related to “Naturalness.” This notion agrees with the intuition that something irregular and elastic is made from natural materials. At present, naturalness is a highly appreciated material property for food and medicine (e.g., Rozin et al., 2004), but only a few studies have examined tactile naturalness as a critical factor in product preference. Further, systematic research is needed to investigate the relationship between the concept of naturalness and tactile perceptual features.

Because the factors extracted from our results were based on data averaged among 60 participants whose age ranged from 19 to 26 years, our results might only reflect the tactile perceptual space of young people. The ability to sense tactile properties is known to decline with aging due to changes in the density and distribution of mechanoreceptors (Stevens and Patterson, 1995). Thus, tactile perceptual space might differ between young and older people. Additionally, differences in skin properties might influence perceptual space. Understanding these types of individual differences in perceptual space is an important issue when designing the tactile properties of products.

In our experiment, we asked participants to rate their impressions of materials and evaluate affective properties through the sense of touch alone (without visual information) because we wanted to examine the representation of tactile perceptual space. However, vision plays an obviously important complimentary role in the perception of objects in our daily lives, and interest in the contributions and interactions of visual and tactile modalities in object perception has been growing. While participants have been reported to be able to retrieve similar information through visual and tactile modalities regarding surface roughness (Bergmann Tiest and Kappers, 2007) and three-dimensional shape (Gaissert and Wallraven, 2012), visual and tactile modalities were shown to be related to distinct perceptual properties in a free-sorting task: shape for vision and substances for touch (Klatzky et al., 1987). Another study has shown that material properties primarily obtained through touch (i.e., hardness and roughness) are crucial for material perception, and the interplay between visual and tactile senses is required for precision (Baumgartner et al., 2013). Our brain is known to integrate visual and tactile information when estimating material properties of an object. When estimating the size (Ernst and Banks, 2002) and the surface roughness (Lederman et al., 1986) of an object through both modalities, these two inputs are averaged. However, visual information can be used to generate prior tactile expectations before touching an object. When they are mismatched, this can cause a contrast effect, such as in the size-weight illusion (Ross, 1969), in which the contrast between expectation and actual percept is emphasized. Investigating visual and tactile perceptual space using SSW is an intriguing direction of future research.

## Conclusion

To obtain a more complete picture of tactile perceptual space, this study focused on sensory vocabulary termed “sound symbolic words” (SSWs) for expressing tactile sensations. We collected a wide range of material samples based on tactile SSWs obtained through testing, and revisited tactile perceptual dimensions. Analysis revealed six major dimensions: “Affective evaluation and Friction,” “Compliance,” “Surface,” “Volume,” “Temperature,” and “Naturalness.” These dimensions include almost all factors mentioned by past studies as well two factors, “Volume” and “Naturalness” that have not been frequently found in other studies. Additionally, we showed that “Affective evaluation” is more closely related to the “Friction” component (slipperiness and dryness) than to other tactile perceptual features. We believe that tactile perceptual dimensions extracted from vocabulary-based analysis will help designers select and create the best range of textures, which is important for obtaining effective affective evaluations.

## Author contributions

MS and JW conceived the experiments. MS performed the experiment and carried out the data analyses. All authors discussed and interpreted the results, and contributed to drafts of this paper.

## Funding

This work was supported by a Grant-in-Aid for Scientific Research on Innovative Areas “Shitsukan” (No. 23135510 and 25135713) from MEXT and a JSPS KAKENHI Grant-in-Aid for Scientific Research on Innovative Areas “Innovative SHITSUKSAN Science and Technology” (No. 15H05922).

### Conflict of interest statement

JW is employed by NTT Communication Science Laboratories, Nippon Telegraph and Telephone Corporation as a research scientist conducting basic scientific research on human sensory processing. There are no patents, products in development or marketed products to declare. This does not alter the authors' adherence to the journal's policies on sharing data and materials. The other author declares that the research was conducted in the absence of any commercial or financial relationships that could be construed as a potential conflict of interest.
