# Supplementary material for: Exploring Tactile Perceptual Dimensions Using Materials Associated with Sensory Vocabulary
Source: Front Psychol. 2017 Apr 13;8:569. doi: 10.3389/fpsyg.2017.00569 (PMC5390040; doi:10.3389/fpsyg.2017.00569)
Supplement: Supplementary file 1 [file Table1.DOCX]

Appendix 1: Details of 120 material samples and corresponding SSWs

| No | Category | Material information | SSWs |
| --- | --- | --- | --- |
| 1 | Particle | Soft wheat flour with wheat as main ingredient | sara-sara  suru-suru |
| 2 |  | Sand (Component: silica, typical surface sand  from Aichi prefecture) | sara-sara  syuri-syuri |
| 3 |  | Glass beads with 1.0 mm diameter particles; made using crushed glass from discarded windows | zasya-zasya  syori-syori |
| 4 |  | Large-grain canary-sand (Component: Calcium carbonate; reused crushed limestone sand from Okayama prefecture) | jari-jari  zasya-zasya |
| 5 |  | Katsura gravel (Component: Silica; gravel obtained from coastal areas in the Philippines) | jori-jori  zaku-zaku |
| 6 |  | Granite (Gravel from granite) | goro-goro  zagu-zagu |
| 7 |  | Glass beads with 3.5 mm diameter particles, Reused crushed glass particles made from discarded windows, etc. | syaka-syaka  tsubu-tsubu |
| 8 |  | Glass beads with 5.0 mm diameter particles, Reused crushed glass particles made from discarded windows, etc. | koro-koro  syaka-syaka |
| 9 |  | Glass beads with 7.0 mm diameter particles, Reused crushed glass particles made from discarded windows, etc. | goro-goro  tsubu-tsubu |
| 10 | Metal  Ceramic  Glass | Aluminum sheet (1000 system) made using standard aluminum with AL purity in excess of 99.0% measuring 3.0 mm (T) × 100 mm (H) × 100 mm (W). | sube-sube  tsuru-tsuru |
| 11 |  | Punched aluminum with surfaces treated by alemite process and pitch of 1Φ×2P and 4000A1000 series materials measuring 0.5 mm (T) × 100 mm (H) × 200 mm (W). | patsu-patsu  suru-suru |
| 12 |  | Punched aluminum with surfaces treated by alemite process and pitch of 1.5Φ×3P and 4000A1000 series materials measuring 0.5 mm (T) × 100 mm (H) × 200 mm (W). | suru-suru  syara-syara |
| 13 |  | Punched aluminum with surfaces treated by alemite process and pitch of 3Φ×4P and 4000A1000 series materials measuring 0.5 mm (T) × 100 mm (H) × 200 mm (W). | poko-poko  tsuru-tsuru |
| 14 |  | Stainless steel with mesh openings of 20 | syori-syori  syusa-syusa |
| 15 |  | Stainless steel with 2.0 mm mesh openings | giza-giza  jori-jori |
| 16 |  | 11-μm-thick aluminum foil | kasa-kasa  gasi-gasi |
| 17 |  | Japanese manufactured ceramic dish with 10 mm diameter particles, thickness of 2.8 cm, and weight of 105 g | goti-goti  tsuru-tsuru |
| 18 |  | Glass measuring 4.0 mm (T) × 99 mm (H) × 99 mm (W). | sube-sube  tsuru-tsuru |
| 19 | Spring | Two units of stainless coil spring with 0.4 mm in length, 6 mm in diameter, and 21 coil windings | gune-gune  unyo-unyo |
| 20 |  | Two units of stainless coil spring with 0.5 mm in length, 7 mm in diameter, and 14 coil windings | den-den  gune-gune |
| 21 |  | A stainless coil spring with 0.7 mm in length, 9 mm in diameter, and 12 coil windings | dan-dan  den-den |
| 22 | Elastomer  Soft-urethane  Gel | Sorbothane, ether-based polyurethane with thickness of 0.3 mm, and impact energy absorption of 94.7% | peto-peto  puni-puni |
| 23 |  | Sorbothane, ether-based polyurethane with thickness of 0.3 mm, and impact energy absorption of 94.7% | peta-peta  puru-puru |
| 24 |  | Styrene elastomer with thickness of 5 mm, 0 hardness, specific gravity of 0.8, and impact energy absorption of over 94.0% | betya-betya  puni-puni |
| 25 |  | Ultra-soft urethane resin for molding with thickness of 1 mm, and 0 hardness | buni-buni  neti-neti |
| 26 |  | Ultra-soft urethane resin for molding with thickness of 2 mm, and 0 hardness | beta-beta  netyo-netyo |
| 27 |  | Ultra-soft urethane resin for molding with thickness of 2 mm, and 5 hardness | beta-beta  nutya-nutya |
| 28 |  | Ultra-soft urethane resin for molding with thickness of 1 mm, and 15 hardness | motyu-motyu  netyo-netyo |
| 29 |  | Ultra-soft urethane gel with thickness of 2 mm, and 15 hardness | beto-beto  peta-peta |
| 30 |  | Ultra-soft urethane gel with thickness of 1 mm, and 30 hardness | peta-peta  peto-peto |
| 31 |  | Ultra-soft urethane gel with thickness of 2 mm, and 50 hardness | peta-peta  punyu-punyu |
| 32 |  | Ultra-soft urethane gel with thickness of 1 mm, and 70 hardness | peto-peto  tsuru-tsuru |
| 33 |  | Semiconductor gel on 100 μm PET substrate with gel-type material on both sides with a thickness of 0.5 mm, and holding strength of 35-40 g / 20 mm | peto-peto  pita-pita |
| 34 |  | Semiconductor gel on 50 μm PET substrate with Mesh M type acrylic pressure sensitive adhesive on both sides with thickness of 0.7 mm, and holding strength of 0-4 g / 20 mm (gel), and 2050 g / 20 mm (acrylic pressure sensitive adhesive) | neti-neti  peto-peto |
| 35 |  | Semiconductor gel on 50 μm PET substrate with dot type acrylic pressure sensitive adhesive on both sides with a thickness of 1.0 mm, and holding strength of 0-1 g / 20 mm (gel), and 2050 g / 20 mm (acrylic pressure sensitive adhesive) | boko-boko  potsu-potsu |
| 36 | Rubber  Leather  Polyure-thane | Rubber with thickness of 10 mm, 55 hardness, and 3 mm deep grooves that supports surface pressure of 2 (kgf / cm2) | kuni-kuni  poko-poko |
| 37 |  | Rubber with thickness of 10 mm, 55 hardness, 10-mm-deep and 10-mm-wide texturing, and 3 mm deep grooves that supports surface pressure of 2 (kgf / cm2) | beko-beko  gyui-gyui |
| 38 |  | Rubber with thickness of 10 mm, 55 hardness, 5-mm-deep and 10-mm-wide texturing, and 3 mm deep grooves that supports surface pressure of 2 (kgf / cm2) | buyo-buyo  gii-gii |
| 39 |  | Rubber with thickness of 10 mm, and attached adhesive sheet with good elastic wear resistance and other mechanical properties | munyu-munyu  suru-suru |
| 40 |  | Foamed polyurethane elastomer with thickness of 5 mm, and vibration absorption in the 40-150 Hz region, load bearing capacity of 40 kg, density of 491 kg / m^3^, static spring constant of 25 N/mm･cm^2^, 25% compressive hardness of 44.1 N/cm^2^, tensile strength of 1.71 MPa, expansion of 150%, tear strength of 4.82 kN/m, and compression set of 4.1% | suru-suru  syuri-syuri |
| 41 |  | Foamed polyurethane elastomer with thickness of 5 mm, and vibration absorption in the 40-150 Hz region, load bearing capacity of 5 kg, density of 155 kg / m^3^, static spring constant of 2.1 N/mm･cm^2^, 25% compressive hardness of 3.9 N/cm^2^, tensile strength of 0.64 MPa, expansion of 167%, tear strength of 2.06 kN/m, and compression set of 8.3% | mugyu-mugyu  suru-suru |
| 42 |  | Chloroprene rubber with thickness of 2 mm: surface used for wetsuits and other purposes | buyo-buyo  hunya-hunya |
| 43 |  | Chloroprene rubber with thickness of 2 mm: the underside used for wetsuits and other purposes | moti-moti  puyo-puyo |
| 44 |  | The surface of cowhide with thickness of 0.8-1.2 mm | gisu-gisu  zuri-zuri |
| 45 |  | The underside of cowhide with thickness of 0.8-1.2 mm | sube-sube  suri-suri |
| 46 |  | Chloroprene rubber with thickness of 1.5 mm | sube-sube  suru-suru |
| 47 |  | Suede with thickness of 0.6 mm made by napping the underside of pigskin. | sara-sara  sawa-sawa |
| 48 |  | Red lizard leather with thickness of 0.5 mm | gisi-gisi  syuri-syuri |
| 49 |  | Sea snake leather with thickness of 0.5 mm | gisi-gisi  zasyu-zasyu |
| 50 |  | Polyester-based urethane foam with cell count 8±2 / 25 mm, density of 30±5 kg / m3, thickness of 10 mm, tensile strength of 49 kPa, and expanded 100% | gesi-gesi  goso-goso |
| 51 |  | Polyurethane with thickness of 10 mm | mosa-mosa  mosyu-mosyu |
| 52 |  | Felt (Wool 60%, rayon 40% with thickness of 6 mm) | suri-suri  wasa-wasa |
| 53 |  | Non-slip net (Three-layer polyvinyl chloride with thickness of 7 mm) | gyui-gyui  muni-muni |
| 54 |  | Non-slip net (PVC coating on two-layer polyester-based net surface with thickness of 4 mm) | gyui-gyui  huni-huni |
| 55 |  | Rubber sheet (Natural rubber 60%) with thickness of 1 mm, and 40 hardness | huni-huni  sube-sube |
| 56 |  | The underside of styrene-butadiene rubber (black) with thickness of 1.5 mm | zara-zara  zuza-zuza |
| 57 |  | Spikey surface (front) of polyester-based hook with thickness of 4.0 mm | jori-jori  tiku-tiku |
| 58 |  | Loop surface (back) of polyester-based hook with thickness of 4.0 mm | syari-syari  syusa-syusa |
| 59 |  | Polyurethane with thickness of 6 mm | butsu-butsu  zara-zara |
| 60 |  | Polyurethane with thickness of 1 mm | zara-zara  zuri-zuri |
| 61 | Paper  Fabric  Clay | Kraft with thickness of 4 mm, paper strength of K5, and fluted C/F | kasa-kasa  kasu-kasu |
| 62 |  | 100% virgin pulp Kraft paper with surface texturing, and thickness of 2 mm | goso-goso  syori-syori |
| 63 |  | Kraft with thickness of 4 mm | suru-suru  syusa-syusa |
| 64 |  | Bark of cork oak with thickness of 5 mm, excellent lightweight, shock-absorbing, elasticity, heat insulating, and water resistant properties | kasa-kasa  syuru-syuru |
| 65 |  | Natural coconut fiber with thickness of 7 mm | kasa-kasa  syari-syari |
| 66 |  | Fine silver streamers | jasi-jasi  wasa-wasa |
| 67 |  | Japanese manufactured polypropylene 100% with thickness of 4 mm, and SBR coating on the back | tiku-tiku  wasa-wasa |
| 68 |  | Special formula EVA resin (ethylene-vinyl acetate copolymer) with thickness of 22 mm | jogi-jogi  zaku-zaku |
| 69 |  | Aluminum, polyethylene, and polyethylene terephthalate | hena-hena  tsuru-tsuru |
| 70 |  | High-purity clay containing inorganic thickener, calcium carbonate, pulp and water, preservative fungicide, and high-quality pulp | nunyo-nunyo  nuru-nuru |
| 71 |  | Soft clay with the consistency of marshmallow that doesn't stick to your hands and is far lighter at 1/8 the weight of ordinary clay consisting of acrylic resin hollow bodies, pulp fiber, synthetic thickener, water, and preservative fungicide | huni-huni  kuni-kuni |
| 72 |  | Nylon (outer packaging), superabsorbent polymer (inner packaging) | bunya-bunya  bunyu-bunyu |
| 73 |  | Silicon rubber containing phosphorescent with thickness of 10 mm | kunyu-kunyu  nyuni-nyuni |
| 74 |  | Japanese manufactured white petrolatum | nuru-nuru  nyupu-nyupu |
| 75 |  | The surface of BCF nylon 100% (pile), soft pack (packing) with thickness of 11.5 mm | mohu-mohu  mosa-mosa |
| 76 |  | The underside of BCF nylon 100% (pile), soft pack (packing) with thickness of 11.5 mm | mosyu-mosyu  syawa-syawa |
| 77 |  | Fire-retardant rubberized formaldehyde (polypropylene 100%) with thickness of 10 mm measures with sound insulation level of LL40 | husa-husa  mowa-mowa |
| 78 |  | Low elasticity urethane foam with shock absorbing and voltage dispersion properties, and thickness of 20 mm | moko-moko  pohu-pohu |
| 79 |  | Polyester-based special cotton 100% and high-resilient cotton for sterilization, odor removal and vacuums | huka-huka  moko-moko |
| 80 |  | Acrylic resin 60% and polyester 40% (front), grand polyester 100% (back), with thickness of 10 mm | mohu-mohu  pohu-pohu |
| 81 |  | Acrylic resin 100% (front), Acrylic resin 55% and polyester 45% (back), with shaggy 10-mm fibers | husa-husa  mohu-mohu |
| 82 |  | Acrylic resin (purple) | husa-husa  mosa-mosa |
| 83 |  | Eight-layer polyester 100% with thickness of 2 mm | suru-suru  syusa-syusa |
| 84 |  | Six-layer nylon 100% with thickness of 2 mm | pera-pera  syusa-syusa |
| 85 |  | Linen (Four-layer 100-thread-count two-ply hemp 100% with thickness of 2 mm) | kasa-kasa  syuri-syuri |
| 86 |  | Four-layer polyester 100% with thickness of 1 mm | kasa-kasa  sara-sara |
| 87 |  | Jeans fabric (Cotton 100% 10-oz denim with thickness of 1 mm) | syuri-syuri  syusa-syusa |
| 88 | Polysty-rene  Rough paper | Urethane foam with hardness No. 4 (hardness ranges from 1-6 with higher numbers indicating increasing hardness) with thickness of 10 mm, and density of 35±3.0 kg / m3 | busyu-busyu  pohu-pohu |
| 89 |  | Polystyrene foam with 60 times expansion ratio, and thickness of 10 mm | kasu-kasu  pusu-pusu |
| 90 |  | Polystyrene foam with 30 times expansion ratio, and thickness of 10 mm | syori-syori  syuwa-syuwa |
| 91 |  | Transparent acrylic sheets that are crimped and packaged in a plastic bag | sube-sube  tsuru-tsuru |
| 92 |  | Sand paper (Silicon carbide particles with No. 600 grain size (finer the meshing the larger the number) with thickness of 1 mm) | gisi-gisi  syori-syori |
| 93 |  | Sand paper (Silicon carbide particles with No. 240 grain size with thickness of 1 mm) | gesi-gesi  gisi-gisi |
| 94 |  | Sand paper (Japanese manufactured three-layer garnet with No. 80 grain size with thickness of 2 mm) | jori-jori  juza-juza |
| 95 |  | Balsa wood manufactured in Ecuador with thickness of 1 mm | sara-sara  syuri-syuri |
| 96 |  | Four-layer high-quality paper with medium thickness, basis weight of 70 g/m2, and thickness of 1 mm | sawa-sawa  sube-sube |
| 97 |  | Single-sided paper with whiteness of 95% (ISO) with special coating enabling rich color printing, and thickness of 0.22 mm | sube-sube  tsuru-tsuru |
| 98 |  | Japanese paper with medium thickness, basis weight of 81.4 g/m2 | sube-sube  syori-syori |
| 99 | Stone | Crystalline limestone, metamorphic rock obtained in Norway with thickness of 30 mm, absorption of 0.13%, compressive strength of 440 kg/cm3, and hardness of 53 Hs | sube-sube  tsuru-tsuru |
| 100 |  | Crystalline limestone, metamorphic rock obtained in Italy with thickness of 30 mm, absorption of 0.10%, compressive strength of 839 kg/cm3, and hardness of 45 Hs | sube-sube  tsuru-tsuru |
| 101 |  | Typical type of gray granite obtained in South Africa with thickness of 30 mm | gasi-gasi  goti-goti |
| 102 |  | Granite with thickness of 30 mm, absorption of 0.16%, compressive strength of 1222 kg/cm3, and hardness of 105 Hs, apparent specific gravity of 2.62, and bending strength of 121.6 kg/cm3 | gasi-gasi  gotsu-gotsu |
| 103 | Others | Kraft with thickness of 20 mm, paper strength of K5, and fluted C/F | boro-boro  gowa-gowa |
| 104 |  | Sawtooth oak with thickness of 20 mm | gotsu-gotsu  zara-zara |
| 105 |  | Polypropylene film with acrylic pressure sensitive adhesive with width of 15 mm | netyo-netyo  nutya-nutya |
| 106 |  | Acrylic high soft form with acrylic pressure sensitive adhesive with thickness of 2.0 mm, and width of 19 mm | neti-neti  petya-petya |
| 107 |  | Scrub brushes (White palm (polypropylene) with thickness of 25 mm) | tiku-tiku  zasyu-zasyu |
| 108 |  | Gel Gems (Type of resin used in Thermoplastic polymer) | puni-puni  puri-puri |
| 109 |  | Chalk (Component: Calcium carbonate; scallop shell powder chalk with 11.2 mm diameter particles) | sara-sara  suri-suri |
| 110 |  | Chalkboard with no one-side wooden frame specs with thickness of 5 mm | kasu-kasu  suru-suru |
| 111 |  | Cotton 100% (2-cm sheet of low-elasticity foam for adjusting height level) | gusyo-gusyo |
| 112 |  | Cloth (slightly moist) (Same as material 78) | gutyo-gutyo |
| 113 |  | Cloth (very moist) (Same as material 78) | syubo-syubo |
| 114 |  | Water | japu-japu |
| 115 |  | Mini slime (Component: Water, guar gum, sodium hydroxide, paraben, boric acid, yellow coloring No. 4, green coloring No. 1) | zupo-zupo  zupu-zupu |
| 116 |  | Polyvinyl alcohol thermoplastic elastomer | toro-toro  yore-yore |
| 117 |  | Methylcellulose mixed with 400 cc of water and 5 g of cellulose | pisya-pisya  pitya-pitya |
| 118 |  | Methylcellulose mixed with 400 cc of water and 10 g of cellulose | nume-nume |
| 119 |  | Add small granules of water to high molecular polymer to expand size by 100-150 times to form one 10-mm-dia grain diameter (hydrolysis state) | putsu-putsu |
| 120 |  | Hokkaido potato starch 100% | poko-poko  sara-sara |
